# Unrecognized Ebola virus infection in Guinea: complexity of surveillance in a health crisis situation: case report

**DOI:** 10.11604/pamj.2020.36.201.24379

**Published:** 2020-07-21

**Authors:** Ibrahima Camara, Mamadou Saliou Sow, Abdoulaye Touré, Bakary Oularé, Elhadj Ibrahima Bah, Salifou Talassone Bangoura, Alioune Camara, Alpha Kabinet Keita

**Affiliations:** 1 Centre de Recherche et de Formation en Infectiologie de Guinée (CERFIG), Université Gamal Abdel Nasser de Conakry, Guinea,; 2 Service des Maladies Infectieuses et Tropicales, Hôpital National Donka, CHU Conakry, Conakry, Guinea,; 3 Chaire de Santé Publique, Département de Pharmacie, Université Gamal Abdel Nasser de Conakry, Conakry, Guinée,; 4 Institut National de la Santé Publique, Conakry, Guinée,; 5 Recherches Translationnelles sur le VIH et les Maladies Infectieuses (TransVIHMI), Institut de Recherche pour le Développement (IRD) UMI 233, Institut National de la Santé et de la Recherche Médicale (INSERM), Université de Montpellier, Montpellier, France

**Keywords:** Ebola, case report, Guinea

## Abstract

The ebola epidemic that raged in West Africa between 2013 and 2016 was the largest since the discovery of the virus in 1976. During this epidemic, more than 11,000 cases were notified with a lethality of over 67%. Several means of transmission have been described. The great difficulty noted during the epidemic was the estimation of the number of asymptomatic and pauci symptomatic cases, however there is evidence that this population has been in contact with the virus for some time. Thus, they could be a source for the spread of the epidemic. In this paper, we report in Guinea-Conakry three stories of probable pauci-symptomatic form of ebola disease that would have been the cause of massive infection in a population sorely tried by the epidemic between 2014 and 2015 in Guinea.

## Introduction

Ebola virus causes serious disease, which is fatal in most of case. The virus infectivity, lethality, the rapidity of human-to-human transmission and the lack of an effective treatment captured the world's attention. The last outbreak in West Africa with 28 652 cases and 11 325 deaths reported, is the largest and most complex ebola outbreak since 1976 [1]. Transmission requires close contact with the body fluids of an infected person during the health care practices, home care or traditional funeral mainly. In Guinea, around 60% of cases have been linked to these practices [1, 2]. Specifically, for this West African ebola virus outbreak, the most affected countries have very weak health systems; also, lack human and infrastructural resources [1]. This lack of capacity has made the control of infections very hard and led to reluctance among affected populations and increased the duration of the outbreak. On August 2014, the WHO declared the West Africa outbreak a public health emergency of international concern under the international health regulations [1]. Drastic measures have been taken in all affected countries for the epidemic control. However, some patients and their contacts sometimes escaped the surveillance system. Either because the system was unable to detect them or because they presented symptomatic or asymptomatic pauci forms.

Indeed, studies conducted near contacts of ebola cases have shown a very high EBOV seroprevalence [1]. In Sierra, Judith R Glyn and her collaborators found an EBOV seroprevalence of 2% in contacts of VME cases. Additionally, 12% of contacts with some symptoms but never diagnosed with ebola virus disease were seropositive. These contacts reported having experienced some symptoms during the active phase of the disease of their indexes [3]. In the contact Ebogui study conducted in Guinea among contacts of VME cases, 3.3% of the contacts had positive EBPV seroprevalence and most of the cases had presented mild symptoms that did not necessitate admission to an ETC during the epidemic [4]. Another study conducted in the DRC in the villages of Kassai in 2017, researchers have an ebola IgB seroprevalence report of 11% [5]. It is estimated that 27.1% of VME cases are asymptomatic [6] and would probably be associated with VMEs that are not linked to an epidemiological chain. In this paper, we report in Guinea-Conakry three stories of probable pauci-symptomatic form of ebola disease that would have been the cause of massive infection in a population sorely tried by the epidemic.

## Patient and observation

### History 1: the reluctance, (Sir LC has 2 wives)

**October 8, 2014:** the first wife infected with ebola virus developed symptoms (fever, abdominal pain, diarrhea and vomiting). She was admitted to the Ebola Treatment Center (ETC) in Conakry. Tested positive for ebola Zaire by RT-PCR. She died the same day. The second wife (MD) and the husband (LC) known as contacts were monitored few days and they escaped the monitoring system.

**October 15, 2014 a week later:** LC has a fever associated with headache and asthenia. Quickly considered as uncomplicated malaria. After self-medication with antimalarial, antibiotic, analgesic and anti-inflammatory drugs, the symptoms disappear in 3 days without ever being admitted to a ETC or healthcare facilities.

**October 28, 2014:** MD, 18 years old, second wife of LC had a headache associated with fever, abdominal pain and vomiting. After 2 days´ self-medication with unknown drugs, she was admitted in a military healthcare facility in Conakry. She was treated for both severe malaria and typhoid fever. Its symptoms disappear after 4 days without tested for ebola virus infection.

**The case that elucidated the history of ebola virus circulation in this family:** MC, 7-month-old (son of LC and MD) has at October 28, 2014, fever associated with diarrhea. He was admitted in the paediatrics service in a Conakry University Hospital. In the first time, he was treated for malaria and acute gastroenteritis. After one week and the occurrence of hematemesis (4 November 2014), he was transferred in Donka ETC and tested positive for ebola virus by RT-PCR but died the same day. The looking for the source of infection has led to perform serology and RT-PCR in parents. They have been positive in ELISA serology and negative in RT-PCR. LC and MD were positive for ebola IgM and IgG antibodies. Despite these medical evidences, the parents remained reluctant and accused the devil. However, a total of 21 individuals were followed by contact tracing teams and none developed symptoms of the ebola virus disease and all were tested negative 2 times in RT-PCR for ebola virus.

### History 2: family of Sir AT burial ceremonies

**On November 7, 2014:** AT, 72 years old have attended the funeral of his son MT; 49 years old (considered as a probable case of infection with the ebola virus).

**On November 19, 2014:** AT developed a fever associated with headache, abdominal pain and joint pain. He has been seen by a physician at the emergency service in an university hospital in Conakry. Known diabetic, her blood sugar was 6.83 mmol/l with a temperature at 39.7°C. He receives antimalarial treatment and an antibiotic. The symptoms disappear after 5 days without ever tested for ebola and without having been admitted to an ETC.

### The case that elucidated the history of the circulation of the virus in this family

**On November 30, 2014:** the AT´s daughter, FT developed a fever over 40°C, diarrhea, vomiting and physical asthenia. She was admitted at the Donka ETC, tested positive for the ebola Zaire virus by RT-PCR. Unfortunately, she died 11 days later. In total, 72 individuals have been registered as contacts around them. They were followed, released after 2 negative PCR and none developed any symptoms related to ebola disease.

**History 3: fear and promiscuity:** AB, 25 years old and his friend ZM 33 years old, are living in the same bedroom.

**On March 26, 2015:** AB: developed a fever associated with headache, dyspnea, joint pain and physical asthenia. After five days, he has been seen by a physician in cardiology department in an university hospital in Conakry. He received as an outpatient antimalarial treatment, antibiotics and anti-inflammatory. Given the persistence of signs, he has been seen by a traditional healer without success. Frightened by the epidemic context, he decided to hide to the contact tracing team. He has been finally located on April 17, 2015; he was asymptomatic. In Donka ETC, he has been tested positive for ebola Zaire by RT-PCR took care there and released after 2 negative consecutive RT-PCR. His friend ZM presented April 3, 2015 a fever, headache and joint pain. He has been admitted to Donka ETC with abdominal pain and diarrhea. Tested positive for ebola virus by RT-PCR. After treatment, he recovered. A total of 36 contacts have been registered monitored and released after 2 negatives PCR and none of them developed any symptoms related to ebola disease.

## Discussion

Zaire ebola virus is the most lethal ebola virus species and one of the most virulent human pathogens, causing a severe hemorrhagic fever syndrome. Fatality rates varied from 25% to 90% in symptomatic patients within a few days [1, 2]. We have reported three particular stories related to ebola disease outbreak in Guinea. Many of the reported cases have gone unnoticed in health facilities. Moreover, in tropical context, it can be difficult to distinguish ebola virus diseases from other infectious diseases such as malaria, typhoid fever. In our situation, thanks to the investigation, we are confident that all the cases described in these stories were symptomatic or pauci-symptomatic forms of the ebola virus disease. Each of them would have been able to expand the Guinea ebola outbreak. All factors were combined for massive infection in these populations. In summary, the reluctance of the population maintained by fear not taking into account certain safety rules and adopting comportements at risk in situation of global health crisis. The situation was worsened by misconceptions around the disease, ignorance of local culture and customs and lack of involvement of local communities in the control strategies. At each step of the development of histories, exposures to a probable contamination were observed at several levels. The health care provider through probably infected patient visits, the general population through direct contact with patients.

In our knowledge, when we are confronted with epidemiological situations of this nature, the analysis of exposure time, the area of the contact and the number of individuals involved is imperative. In our scenario, we have every reason to believe that through these stories, the epidemic could have been being restarted with no possibility to control. However, some weaknesses in the contact tracing system established, people were finally identified, confined and treated. These stories show once again the difficulties that may be encountered for the control of such an epidemic. These stories raise the question of the sexual transmission of the virus [7]. In the first story, the man probably contaminates his wife during sexual relation. It is now proven that the virus can be transmitted through sexual relation [7]. The persistence of virus in semen could explain this situation [7, 8]. Recent studies have shown evidence sexual transmission related to the persistence of ebola virus in semen [7-9]. The possibility of mother-to-child transmission through the persistence of the virus in breast milk was recently raised [10]. The big question that researchers have always tried to answer is how many asymptomatic ebola cases have we recorded and can asymptomatic people transmit the disease. If for the first question extensions are available for the second question no data was found in the literature. Thus, we report in this manuscript a possible case of transmission from an asymptomatic mother to her nursing child.

## Conclusion

These three case reports raise the problem of investigating and monitoring ebola cases in a health emergency context, but also raise the question of the role of asymptomatic ebola cases in the spread of the disease. It is obvious that there have been shortcomings in the management of the epidemic in Guinea and that not all the exhaustive number of cases is known.

**Ethical aspects:** this study was sponsored by the ethics committee of the public health department of the Abdel Nasser Gamal University in Conakry. Data were collected as part of response activities against the ebola outbreak in Guinea and personal information was not disclosed in this article to protect the anonymity of patients.
